# Supplementary material for: Automatic Knee Osteoarthritis Diagnosis from Plain Radiographs: A Deep Learning-Based Approach
Source: Sci Rep. 2018 Jan 29;8:1727. doi: 10.1038/s41598-018-20132-7 (PMC5789045; doi:10.1038/s41598-018-20132-7)
Supplement: Supplementary file 1 — Supplementary information [file 41598_2018_20132_MOESM1_ESM.pdf]

Automatic Knee Osteoarthritis Diagnosis from Plain Radiographs: A Deep Learning-Based Approach

Aleksei Tiulpin[1,*], **Jérôme Thevenot**[1], **Esa Rahtu**[3], **Petri Lehenkari**[2], **and Simo Saarakkala**[1,4]

[1]Research Unit of Medical Imaging, Physics and Technology, University of Oulu, Finland
[2]Department of Anatomy, University of Oulu, Finland
[3]Department of Signal Processing, Tampere University of Technology, Tampere, Finland
[4]Department of Diagnostic Radiology, Oulu University Hospital, Oulu, Finland
[*]aleksei.tiulpin@oulu.fi

1 Model selection process and comparison to the previous work

In our implementation, we used the PyTorch framework and 4×NVidia GTX1080 cards. Because the MOST dataset was very imbalanced because of the low presence of higher KL grades, we used oversampling to overcome this issue in all our experiments: for each training epoch, we randomly sampled with repetitions roughly $N_{cat} \times B$ images from each of the categories (KL 0–4), where N_{cat} is the average number of training examples per category in our training set and B is a bootstrap factor. From our training data we found $N_{cat} \times B = 3,675$. Parameter $B = 15$ was found empirically by trial and error. We found this strategy useful to prevent overfitting, especially when it is combined with data augmentation and selection of the number of batches per epoch (Table 1). For data augmentation, we used brightness, contrast, rotation, gamma correction and jitter. In our experiments, we mostly used Adam's method with a learning rate of $1e-2$; however, to reproduce the results presented by Antony et. al. in[1], we used a stochastic gradient descent with Nesterov momentum and learning rate of $1e-4$. The batch size which was used in all our experiments was empirically selected to be 64.

We systematically compared multiple models — multiple configurations of our proposed approach. First, we re-implemented the best-performing network described in the article by Antony et al. This network produces two outputs — one for classification and the other one for regression. The optimisation is done by minimising an average of mean squared error (MSE) and cross-entropy. The idea behind this loss function is to give a network information about the importance of higher (e.g., KL4) versus lower (KL0) misclassifications. In our implementation, we cropped the 300×300 to 300×200 pixel images and used them as the network input, as described in the manuscript. The 300×300 images were obtained after the data augmentation. Due to the insufficient implementation details provided in the original paper and the differences in our training settings, we could not exactly reproduce the results; however, we found validation performance in the multi-class average accuracy and MSE that were similar to the values reported by the authors. To achieve these results, we had to use the following strategy: starting from the learning rate of $1e-4$ we were dropping 10 times it each 50,000 iterations. When the learning rate drop was less than $1e-6$, we increased it back to $1e-4$ and continued this procedure. In total we trained the network for 250,000 iterations. This was performed because of the plateau in training, and it helped to escape the achieved local minima.

Secondly, we performed a fine-tuning of a ResNet-34 network[2] that was pre-trained on the ImageNet dataset. We found this model overfitting quickly so decided to evaluate it more frequently – 300 iterations per training epoch compared to our model and the model from Antony *et al.*[1] – 500 iterations per training epoch. In total, we trained ResNet-34 for 14, 300 iterations and had to stop the process afterwards because the validation loss started to rapidly increase. We summarise all our results in Table 1. Based on the validation Kappa, we selected the fine-tuned ResNet and our model with $N = 64$ for a qualitative comparison, as described in the article and the next section.

Table 1. Model selection and comparison to the other models. Here, N in the own models indicates the number of filters in the first layer, as in the main text of the article, and indicates whether the weights of the network branches were shared or not. # Batches indicates the epoch size, Kappa corresponds to the quadratic Kappa coefficient, MSE to the mean squared error and Accuracy the average multi-class accuracy. All the models were trained with a batch size of 64 samples. Column Kappa shows in bold the two best models – our models with the starting number of filters $N = 64$ and the fine-tuned ResNet-34.

Model	Learning rate	# Batches	Optimizer	Kappa	MSE	Accuracy
Own [N=32]				0.803	0.526	67.04
Own [N=32, NS]				0.706	0.732	56.40
Own [N=64]	$1e-2$	500		**0.808**	0.518	64.77
Own [N=64, NS]			Adam	0.718	0.736	57.81
Own [N=128]				0.801	0.515	66.35
Own [N=128, NS]				0.727	0.705	58.78
ResNet-34	$1e-3$	300		**0.812**	0.512	67.02
Model by Antony *et al.*, 2017	$1e-4$	500	SGD	0.770	0.670	59.52

2 Attention maps and probability distribution examples

In this section, we present examples of the attention maps produced by the fine-tuned ResNet-34 and our model for clinically relevant cases KL-2 (Figure 1) and also for already present, moderate OA (Figure 2). The attention maps indicate the benefit of constraining the attention of the network by using prior anatomical knowledge.

References

1. Antony, J., McGuinness, K., Moran, K. & O'Connor, N. E. Automatic detection of knee joints and quantification of knee osteoarthritis severity using convolutional neural networks. *arXiv preprint arXiv:1703.09856* (2017).
2. He, K., Zhang, X., Ren, S. & Sun, J. Deep residual learning for image recognition. In *Proceedings of the IEEE Conference on Computer Vision and Pattern Recognition*, 770–778 (2016).

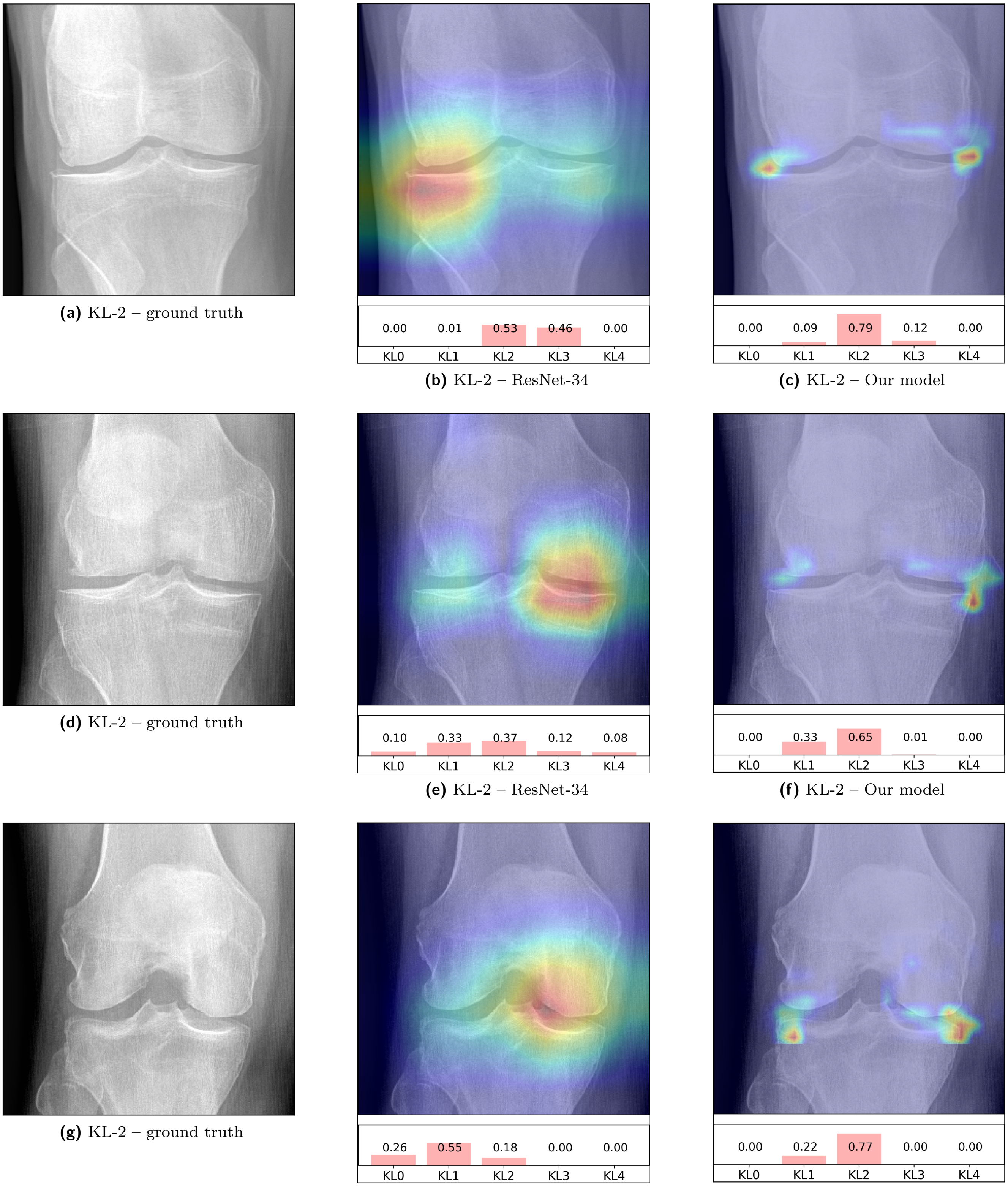

(a) KL-2 – ground truth

(b) KL-2 – ResNet-34

(c) KL-2 – Our model

(d) KL-2 – ground truth

(e) KL-2 – ResNet-34

(f) KL-2 – Our model

(g) KL-2 – ground truth

(h) KL-1 – ResNet-34

(i) KL-2 – Our model

Figure 1. Comparison of the attention maps and output probability distributions between the baseline and our method for the clinically relevant case KL-2. The examples show that the pre-trained model is less certain than our proposed approach.

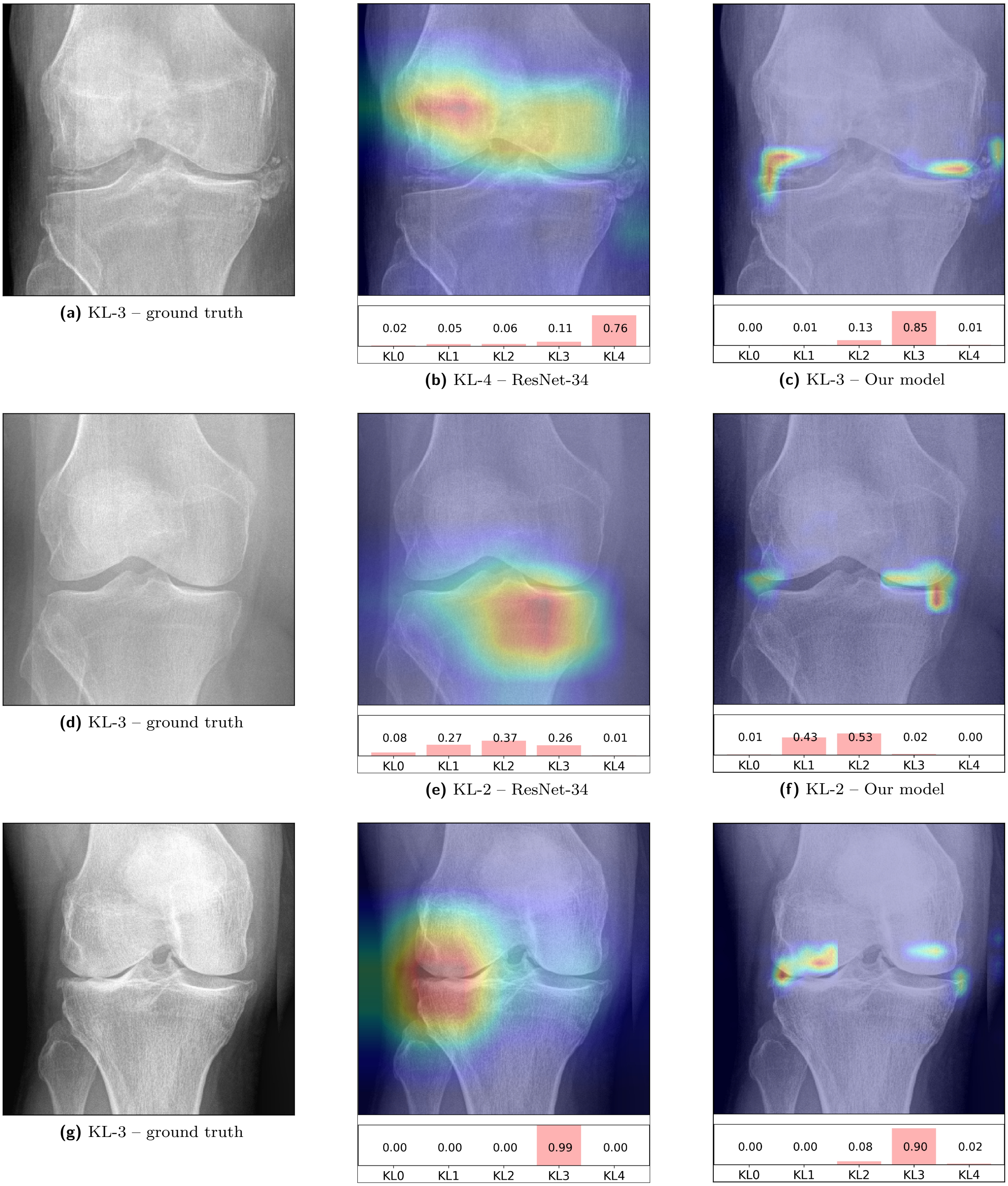

(a) KL-3 – ground truth **(b)** KL-4 – ResNet-34 **(c)** KL-3 – Our model

(d) KL-3 – ground truth **(e)** KL-2 – ResNet-34 **(f)** KL-2 – Our model

(g) KL-3 – ground truth **(h)** KL-3 – ResNet-34 **(i)** KL-3 – Our model

Figure 2. Comparison of the attention maps and output probability distributions between the baseline and our method for detection of moderate osteoarthritis (KL-3).
